# The Treatment of Ingrown Nail: Chemical Matricectomy With Phenol Versus Aesthetic Reconstruction. A Single Blinded Randomized Clinical Trial

**DOI:** 10.3390/jcm9030845

**Published:** 2020-03-20

**Authors:** Juan Manuel Muriel-Sánchez, Ricardo Becerro-de-Bengoa-Vallejo, Pedro Montaño-Jiménez, Manuel Coheña-Jiménez

**Affiliations:** 1 Facultad de Enfermería, Fisioterapia y Podología, Universidad de Sevilla, 41009 Sevilla, Spain; murielsanchezjm@gmail.com (J.M.M.-S.); pmj@us.es (P.M.-J.); 2 Facultad de Enfermería, Fisioterapia y Podología, Universidad Complutense de Madrid, 28040 Madrid, Spain; ribebeva@ucm.es

**Keywords:** Ingrown nail, chemical matricectomy, aesthetic reconstruction, surgery

## Abstract

Background: In onychocryptosis surgery, incisional and non-incisional matricectomy is indicated according to the stage. The chemical matricectomy with 88% phenol solution is the gold standard and a wedge resection is indicated for more advanced stages. The aesthetic reconstruction has the advantages of the incisional procedure without eponychium incisions and an effectiveness similar to the chemical matricectomy with phenol. Objective: To compare the recurrence and the healing time between the chemical matricectomy with phenol and the aesthetic reconstruction. Methods: A comparative, prospective, parallel, randomized, and one-blinded clinical trial was registered with the European Clinical Trials Database (EudraCT) with identification number 2019-001294-80. Thrity-four patients (56 feet) with 112 onychocryptosis were randomized in two groups. Thirty-six were treated with chemical matricectomy with phenol and 76 with aesthetic reconstruction. Each patient was blind to the surgical procedure assigned by the investigator. The primary outcome measurements were healing time and recurrence. The secondary outcome measurements were post-surgical bleeding, pain, inflammation, and infection rate. Results: The aesthetic reconstruction procedure presents a shorter healing time (8.2 ± 1.4 days vs. 21.3 ± 3.1 days; *p* < 0.001) with a similar recurrence rate (*p* = 0.98). Post-operatory bleeding, pain, inflammation, and the infection rate did not show significant differences (*p* > 0.05). Conclusions: The aesthetic reconstruction presents a shorter healing time, favoring the patients’ recuperation, with a recurrence similar to the chemical matricectomy with 88% phenol solution.

## 1. Introduction

Onychocryptosis is a pathologic condition of the nail apparatus in which the toenail damages the nail fold. It is a common condition provoking pain, inflammation, and functional limitation [1]. There are numerous surgical procedures and modifications described in the surgery of ingrown nail [1,2,3,4,5], its causes, and contributing risk factors [6]. There exist various non-incisional surgical options, such as the gold standard procedure, chemical matricectomy with 88% phenol solution, and its modifications [7,8,9], such as the modification which eliminates cauterized tissue via thorough curettage [1], or different duration for the application time of phenol [10].

Local application of phenol solution may be considered as the first non-incisional surgical option with a recurrence rate of less than 5% [11]. This option shows beneficial effects, such as strong antiseptic properties, production of necrosis during protein coagulation, and reduction of pain due to nerve fiber demyelination in the nail unit [11,12]. Nevertheless, chemical matricectomy with 88% phenol solution may produce a delay in the healing time (between 21 and 42 days) related to tissue destruction, excessive drainage, and an adverse reaction to the phenol solution [10,13,14,15]. On the other hand, incisional surgical options are indicated for advanced stages with hypertrophy of the nail folds. Winograd procedure may be considered as the first incisional surgical option [1,3].

Some non-incisional surgical options do not use chemical agents and reduce the post-operatory time, as the aesthetic reconstruction [1], which offers a better cosmetic result of the nail. First, a wedge resection of soft tissue is carried out, eliminating the germinal cells of the matrix and of the nail bed, and later a curettage is done of the surgical zone. The chemical matricectomy with phenol has the highest effectiveness rate [16], but it has a long healing time [7,8,9,10,11,12,13,14,15]. Aesthetic reconstruction has as advantages, a shorter healing time with respect to chemical matricectomy with phenol with a similar effectiveness [1]. No cutaneous incision is made, and therefore no stitches are required.

Consequently, the aim of this study was to compare the aesthetic reconstruction and chemical matricectomy with 88% phenol solution which are commonly used surgical options in terms of healing time and recurrence rate. Secondly, we suggested contrasting the effect that both procedures have on the result indicators: post-surgical bleeding, pain, inflammation, and the infection rate.

## 2. Materials and Methods

### 2.1. Design and Sample 

A comparative, prospective, parallel, randomized, one-blind clinical trial was carried out according to the Consolidated Standards of Reporting Trials (CONSORT) guidelines [17]. The study sample were patients treated in the Clinical Area of Podology of the University of Seville in Spain and took place between June 2017 and March 2018. The participants gave their written consent and were monitored for 8 months. Thirty-four healthy participants with onychocriptosis (n = 56 halluces; 112 nail folds) had previously been given conservative treatment without definitive results. The patients were submitted to experimental conditions (simple randomization: flipping a coin); Heads = 36 nail folds with the chemical matricectomy with phenol (10 patients; 18 nails) and Tails = 76 nail folds with aesthetic reconstruction (24 patients; 38 nails). The same researcher generated the random allocation sequence, they enrolled participants, and assigned participants to interventions. Each patient was blind to the surgical procedure assigned by the investigator.

The inclusion criteria were to have ingrown nail in stages I or IIa [1], to be older than 18 years, to be of either sex, not having an underlying bone pathology, an indication of partial matricectomy. The exclusion criteria were to have chronic illnesses, serious circulatory problems, badly controlled diabetes, to be pregnant, having wound healing disorders, to be sensitive to phenol, or to have had previous ingrown nail surgery. The result parameters were recurrence and the healing time in relation to the procedure used. The secondary result parameters were bleeding, post-operatory pain, inflammation, and the infection rate.

### 2.2. Surgical Procedures

**Figure 1:**
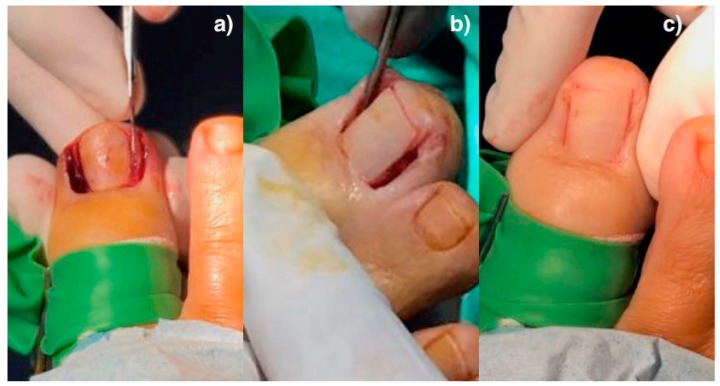
Aesthetic reconstruction: (**a**) Subeponychial wedge excision; (**b**) curettage of the matrix zone and the nail bed; (**c**) coapting the folds.

For the aesthetic reconstruction group, the surgical procedure consisted in the partial ablation of the nail associated with an aesthetic reconstruction of the nail fold [1]. First, a digital blocking of the hallux using 2% of Mepivacaina. Afterward, a digital tourniquet was done for the local hemostasis of the hallux. The nail plate affected was separated from the nail bed and the eponychium. The first cut was with a cutter and afterward with a nº 15 scalpel. Next, the fragments of the nail plate were extracted. In the aesthetic reconstruction technique, the nail fragment is ablated and then the soft tissue wedge is removed, without making incisions in the skin, all at the subeponychial level. A wedge excision of the soft tissue, including the nail matrix and the bed of the portion of tissue affected, was done, resecting out the edges of the hypertrophic folds was necessary (Figure 1a).

To check the intraoperative result of the excision, a coaptation of the folds of the nail was carried out and subsequently, a careful curettage of the matrix zone and the nail bed (Figure 1b). Coapting the folds means to approximate them with the help of the surgeon´s fingers to check the result of soft tissue excision (Figure 1c). After scrubbing with physiological saline with pressure to drag the biological remains, the nail folds were constructed with the help of approximation strips. The surgical wound was covered with non-stick absorbent sterile polypropylene, impregnated with antiseptic, and a partial bandage of the hallux was put in place. The hemostasis was removed, the blood test was checked, and a definitive compressive bandage was placed on the hallux.

For the chemical matricectomy with the phenol group, after removing the portion of the nail plate, a swab with a cotton ball soaked in 88% phenol was applied for 1 minute to the zone of the matrix and the nail bed [7]. This zone was irrigated with 76% ethanol for one minute and then with a physiological saline solution. The surgical wounds were covered with a thin layer of sulfadiazine silver cream and after, with non-stick absorbent sterile polypropylene. Gauze was placed around the hallux and covered with a sterile compressive bandage. 

### 2.3. Outcome Measurements

The same clinic carried out all the surgical procedures of the hallux and the other clinic did the mediators of the study variables with a clinical and photographic follow-up. The surgical verification list was used to improve the patients’ safety [18].

The primary outcome measurements were healing time and recurrence rate. The healing time was measured paying attention to the previously described criteria [2,19], considering it to be the period of time between the surgical action and the solving of the draining and/or inflammatory changes. These criteria are absence of exudate in the gauze; the forming of a scab which covers the granulation tissue; the wound must be kept uncovered; a lack of signs of infection or inflammation in the zone operated; there are no signs of erythematosus tissue or of hypergranulation. To measure recurrence, a relapse of clinical reappearance during a follow-up of a minimum of six months was considered [16]. Likewise, the growth of an asymptomatic nail spicule was regarded as a post-operatory sequel and not as a recurrence [20].

The secondary outcome measurements were post-surgical bleeding, pain, inflammation, and infection rate. The intensity of the bleeding came from the photographic assessment carried out during the first dressing and classified (light, moderate, abundant) by two other researchers. The bleeding indicator was classified as mild when this partially stains the cellulose dressing and the gauze, as moderate when it totally stains the dressing and partially the gauze in contact, and abundant when it stains the dressing and much of the gauzes, and the bleeding is visible [2]. Pain indicator was measured 24/48/72 hours after the surgery via a 10 cm visual analogical scale (0 = without pain; 10 = maximum pain). Post-surgical inflammation as measured by the digital circumference in mm using a flexible ruler (Devon Industries 1-800, Inc., Devon, PA, USA) at the level of the proximal nail fold before and 48h after surgery during the acute inflammatory phase of healing [2,11]. Presence of infection was considered when there was pain and clinical drainage, or pus secretion with erythema was noted [12]. 

### 2.4. Sample Size Calculation

The sample size required for the study was calculated using CTM-1.1 (Glaxo Wellcome SA, Madrid, Spain). The result of the computation was that, to detect a clinically relevant difference of 8 days [1,7] in mean healing time between the groups. Considering a two-tailed test, an α error of 0.05, a desired power of 80% (β = 20%) and estimating a follow-up loss rate of 10%, a minimum sample size of 26 nail folds per group was considered.

### 2.5. Ethical and Legal Considerations

The clinical trial was registered in the Australian New Zealand Clinical Trials Registry (trial id: ACTRN12619000399190) and the European Clinical Trial Database (EudraCT id: 2019-001294-80). The research was also approved by the Bioethics Committee of the Government of Andalusia (id: 1861-N-17) and authorized by the Head Office of the Clinical Area of Podiatry of the University of Seville (id:I NVO4-18). Moreover, the guidelines associated with the ethical standards for investigation and experimentation in human participants as reported in the Declaration of Helsinki at the 64th World Medical Assembly (Fortaleza, Brazil) and other international institutional organizations were maintained.

### 2.6. Statistical Analysis

The qualitative variables were expressed through their frequencies and percentages; the quantitative variables in averages and standard deviations, and 95% confidence interval (CI; lower and upper limits) for parametric data in addition to median and interquartile range (IR) for non-parametric data. The Kolmogorov–Smirnov test (aesthetic reconstruction) and Saphiro–Wilk test (chemical matricectomy) for normality were applied. The healing time, recurrence, and infection were measured by nail folds and the bleeding and pain variables by toes. The Chi-square test with Yates continuity correction was applied to the contingency tables two by two of the recurrence and infection variables for the comparison between the groups. The Mann–Whitney U test for non-normal distributions compared the healing time, pain, inflammation, and bleeding variables or t student independent for normal distribution. SPSS 22.0. (SPSS, Inc., Chicago, IL, USA) was used for the statistical analysis, and statistically significant differences were set at *p* < 0.05 with a 95% CI.

## 3. Results

### 3.1. Descriptive Data

**Figure 2:**
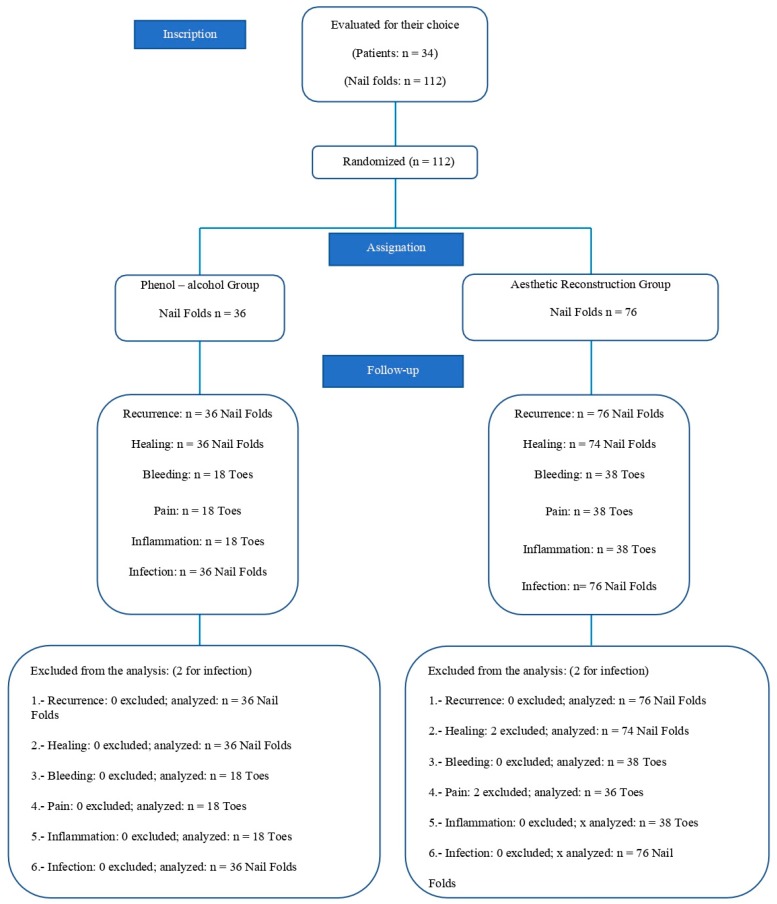
Flow diagram of patients throughout the course of the study.

A total of 34 patients, 12 men and 22 women, and 56 feet (112 nail folds) were registered, performed during 2017–2018 (Figure 2).

The average age of the sample was 34.0 years (S.D. = 18.290). Thirty-six (32.1%) of the nail folds were of the chemical matricectomy group and 76 (67.9%) of the aesthetic reconstruction group. Fifty-six halluces—38 (67.9%) for the aesthetic reconstruction procedure and 18 (32.1%) for that of chemical matricectomy. In addition, 60.7% (n = 68) belonged to women and 39.3% (n = 44) to men. The characteristics of the study patients for each group are represented in Table 1.

### 3.2. Outcome Measurements

The healing time and recurrence were the primary indicators described before. For the chemical matricectomy group, the healing time was 21.3 ± 3.1 days; the aesthetic reconstruction group 8.2 ± 1.4 days. The difference was statistically significant (Mann–Whitney U-test: *p* = 0.000). The recurrence rate was 2.8% for the chemical matricectomy group and 1.52% for the aesthetic reconstruction group, the difference not being statistically significant (Yates’ Chi-square test = 0.98). The results of the secondary indicators did not show statistically significant differences between the groups. The infection rate was higher for the chemical matricectomy group (Yates’ Chi-square test = 0.82). Pain was greater after 24/48/72 hours for the aesthetic reconstruction group. The results of the variables are given in Table 2.

## 4. Discussion 

This clinical trial means to compare one of the chemical procedures most noted in the literature and applied in clinical practice [12], chemical matricectomy with phenol, with the aesthetic reconstruction procedure [1], with the aim of clarifying its preference in nail surgery. Systematization being necessary based on the evidence according to different clinical presentations.

Our results show significant differences in the healing time, favorable for the aesthetic reconstruction group, addressing the healing criteria used in previous clinical trials [2]. Vaccari et al. obtained a healing time of between 14–28 days in partial phenolization [21]. In our study, this time was 8 days for the aesthetic reconstruction group and 20.5 for the chemical matricectomy group. According to our results, the chemical matricectomy requires a longer healing time than aesthetic reconstruction, this having been reduced to 8 days and with better results than other authors, who reported 13 days [1,22].

Another study shows a healing time of 21 days in chemical matricectomy [23], with results similar to our study and other retrospective investigation [24]. This was reduced to 7 days in another study in the chemical matricectomy group [25], whose criterion was the relief of symptoms and the capacity to recuperate daily life activities. The time increased to 2.1 weeks in a comparative clinical trial using wedge resections with partial phenolization [26]. In contrast, other authors have compared segmental phenolization with surgical resection of the matrix without finding significant differences between the healing times [27]. On the other hand, another study showed that operation time was fairly short in chemical matricectomy with NaOH in comparison to the wedge resection method [28].

Although studies do not exist that are similar to this one, in order to be able to establish complete comparisons, our results demonstrate a high effectiveness in both procedures. Another study obtained recurrence rates of 20.6% after the wedge resection [27]. We have considered effectiveness as being the assessable recurrence rate 6 months after surgery [12]. Another investigation [29] obtained higher recurrence rates (1.1%–5.7%). Our results were 2.8% in the chemical matricectomy group and 1.52% in the aesthetic reconstruction group, these results agree with Álvarez et al. (2012) in their clinical trial [2] and Karaka and Dereli (2012) of 99.7% with a longer follow-up period [30].

We believe that a high recurrence rate is due to not doing a matricectomy, so we carried this out on all the patients. Hassel et al. (2010) reported a recurrence rate of 31.5% for the chemical matricectomy group and 6.9% for the surgical matricectomy [25]. These results are contrary to reported by others [31]. In line with this, a chemical procedure has been compared with surgical procedure reporting low rates of recurrence or infection [32].

To analyze the bleeding indicator, it must be taken into account that the procedures compared were carried out under conditions of ischemia. We did not obtain significant differences between the two procedures, differing from other studies where the bleeding was greater both in the surgical procedure and in the chemical procedure [2], and which established a correlation between the increase of the bleeding and a longer healing time. Our results show non-significative differences between both groups for the bleeding indicator. 

According to the literature on pain [22,25,27], this was greater after 24/48/72 hours for the aesthetic reconstruction group. However, statistically significant differences between the two were not shown. Another clinical study reported partial matricectomy with electrocautery prolonged duration of pain and inflammation [33].

Another aim of this clinical study was to compare the post-operative infection rates as a clinical indicator of post-operative recuperation: no significant differences were observed between the groups. The aesthetic reconstruction presented a 1.5% infection rate and chemical matricectomy, 5.6%. In line with literature, the preoperatory antibiotic prophylaxis of the ingrown nail did not use it [32]. Previous studies reported the presence of exudate in their surgical wounds after 2–4 days, being prolonged up to 30 days [27]. On the contrary, others did utilize it, obtaining a low infection rate [2].

Aesthetic reconstruction is an effective procedure, without the need to use a chemical agent. Therefore, this study shows the procedure as an alternative with a low recurrence rate and with a significantly shorter healing time.

The study was limited due to the bleeding and pain indicators having been evaluated according to the affecting toe. This fact could be resolved by designing a study evaluating only one nail fold. This study also presented as a limitation the long-term follow-up period; patients did not attend the final 12-month review. 

## 5. Conclusions

This randomized controlled clinical trial has demonstrated that the aesthetic reconstruction procedure offers a shorter healing time, favoring the patients’ recuperation, without there being significant differences in the recurrence rates. 

## Figures and Tables

**Table 1:** Characteristics of the study sample by sex distribution.

Characteristics	Chemical Matricectomy with Phenol	Aesthetic Reconstruction Group	Total Analyzed
Patients	10 (29.4%)	24 (70.6%)	34 (100%)
Males	4	8	12 (35.3%)
Females	6	16	22 (64.7%)
Feet	18	38	56
Nail Folds	36 (32.1%)	76 (67.9%)	112 (100%)
Average Age	37.1	32.4	34.0
SD	20.3	17.7	18.3

Abbreviations: SD, standard deviation. The average age is expressed in years.

**Table 2:** Comparisons for outcome measurements between both treatment groups.

Outcome Measurements	Chemical Matricectomy with Phenol	Aesthetic Reconstruction Group	*p*-Value
	Mean ± SD (95% CI) Median (IR)	Mean ± SD (95% CI) Median (IR)	
Healing time (days)	21.3 ± 3.1 (20.20–22.39) 20,5 (11)	8.2 ± 1.4 (7.92–8.55) 8 (7)	<0.001 *
Recurrence rate	1 (2.8%) **	2 (1.52%) **	0.98 ***
Post-surgical bleeding (Mild = 1; moderate = 2; abundant = 3)	1.67 ± 0.58 (1.48–1.86) 2 (1)	1.65 ± 0.62 (1.51–1.79) 2 (1)	0.91 *
Post-surgical pain at 1st day (VAS)	1.9 ± 1.8 (1.31–2.49) 1.5 (3)	2.6 ± 2.5 (2.04–3.16) 2 (4)	0.41 *
Post-surgical pain at 2nd day (VAS)	1.2 ± 1.4 (0.74–1.66) 1 (2)	1.9 ± 2.2 (1.41–2.39) 1 (4)	0.28 *
Post-surgical pain at 3rd day (VAS)	0.8 ± 1.2 (0.41–1.19) 0 (1)	1 ± 1.3 (0.71–1.29) 1 (2)	0.33 *
Post-surgical inflammation (mm; digital circumference)	0.2 ± 0.1(0.17–0.23) 0.2	0.3 ± 0.3 (0.23–0.37) 0.3	0.72 *
Infection rate	2 (5.6%) **	2 (1.5%) **	0.82 ***

Abbreviations: CI, confidence interval; IR, interquartile range; SD, standard deviation; VAS, visual analogue scale. * Mann–Whitney U test. Statistically significant differences were set at *p* < 0.05 with a 95% CI. ** Qualitative variable; frequencies (percentage). *** Yates’ Chi-square test.

## References

[1] Martínez-Nova A., Sánchez-Rodríguez R., Alonso-Peña D. (2007). A new onychocryptosis classification and treatment plan. J. Am. Pod. Med. Assoc..

[2] Álvarez J., Córdoba-Fernández A., Munuera P.V. (2012). Effect of curettage after segmental phenolization in the treatment of onychocryptosis: A randomized double-blind clinical trial. Dermatol. Surg..

[3] Winograd A. (2007). A modification in the technic of operation for ingrown toenail. J. Am. Pod. Med. Assoc..

[4] Cologlu H., Kocer U., Sungur N., Uysal A., Kankaya Y., Oruç M. (2005). A new anatomical repair method for the treatment of ingrown nail. Annals Plastic. Surg..

[5] Zhang N., Huang Z., Cao S.H., Wang Y., Hu Y. (2018). Cosmetic, minimally invasive, partial matricectomy of ingrown toenails with granulation tissue. J. Plast. Reconstruc. Aesth. Surg..

[6] Córdoba-Fernández A., Montaño-Jiménez P., Coheña-Jiménez M. (2015). Relationship between the presence of abnormal hallux interphalangeal angle and risk of ingrown hallux nail: A case control study. BMC Musculoskelet. Disord..

[7] Boll O. (1945). Surgical correction of ingrowing nails. J. National Assoc. of Chir..

[8] Córdoba-Díaz D., Becerro-de-Bengoa-Vallejo R., Losa-Iglesias M.E., Córdoba-Díaz M. (2014). Effectiveness of standard lavage with supplemental chlorhexidine in patients undergoing chemical matricectomy for ingrown toenails: A clinical trial. J. Am. Acad. Dermatol..

[9] Dika E., Balestri R., Vaccari S., Alessandro P., Misciali C., Patrizi A. (2009). Successful treatment of pyogenic granulomas following gefitinib therapy with partial matricectomy and phenolization. J. Dermatol. Treat..

[10] Becerro-de-Bengoa-Vallejo R., Losa-Iglesias M.E., Viejo-Tirado F., Serrano-Pardo R. (2012). Cauterization of the germinal nail matrix using phenol applications of differing durations: A histologic study. J. Am. Acad. Dermatol..

[11] Garrido-Castells X., Becerro-de-Bengoa-Vallejo R., Calvo-Lobo C., Losa-Iglesias M.E., Palomo-López P., Navarro-Flores E., López-López D. (2019). Efectiveness of leukocyte and platelet-rich fibrin versus nitrofurazone on nail post-surgery bleeding and wound cicatrization period reductions: A randomized single blinded clinical trial. J. Clin. Med..

[12] Rounding C., Bloomfield S. (2005). Surgical treatments for ingrowing toenail. Cochrane Database Syst. Rev..

[13] Becerro de Bengoa Vallejo R., Losa Iglesias M., Alou Cervera L., Sevillano Fernaández D., Prieto Prieto J. (2010). Total nail ablation for onychodystrophy with optimized gauze-phenol application. J. Eur. Acad. Dermatol. Venereol..

[14] Iglesias M.E.L., De bengoa vallejo R. (2010). Topical Phenol as a Conservative Treatment for Periungual Pyogenic Granuloma. Dermatol. Surg..

[15] Becerro de Bengoa Vallejo R., Losa Iglesias M.E., Sanchez Gomez R., Jules K.T. (2008). Gauze application of phenol for matrixectomy. J. Am. Podiatr. Med. Assoc..

[16] Eekhof J., Van-Wijk B., Knuistingh-Neven A., van-der-Wouden J.C. (2012). Interventions for ingrowing toenails. Cochrane Database Syst. Rev..

[17] Begg C., Cho M., Eastwood S. (1996). Improving the quality of reporting of randomized controlled trials. The CONSORT statement. J. Am. Med. Assoc..

[18] García-París J., Coheña-Jiménez M., Montaño-Jiménez P., Córdoba-Fernández A. (2015). Implementation of the WHO Safe Surgery Saves Lives checklist in a podiatric surgery unit in Spain: A single-center retrospective observational study. Patient Saf. Surg..

[19] Córdoba-Fernández A., Rayo-Rosado R., Juarez-Jiménez J. (2008). Platelet gel for the surgical treatment of onychocryptosis. J. Am. Pod. Med. Assoc..

[20] Ozawa T., Nose K., Harada T., Muraoka M., Ishii M. (2005). Partial matricectomy with a CO_2_ laser for ingrown toenail after nail matrix staining. Dermatol. Surg..

[21] Vaccari S., Dika E., Balestri R., Rech G., Piraccini B.M., Fanti P.A. (2010). Partial excision of matrix and phenolic ablation for the treatment of ingrowing toenail: A 36-month follow-up of 197 treated patients. Dermatol. Surg..

[22] Persichetti P., Simone P., Li-Vecchi G., Di-Lella F., Cagli B., Marangi G.F. (2004). Wedge excision of the nail fold in the treatment of ingrown toenail. Annals Plast. Surg..

[23] Shaath N., Shea J., Whiteman I., Zarugh A. (2005). A prospective randomized comparison of the Zadik procedure and chemical ablation in the treatment of ingrown toenails. Foot Ankle Int..

[24] Bostanci S., EkmekçI P., Gürgey E. (2001). Chemical matricectomy with phenol for the treatment of ingrowing toenail: A review of the literature and follow-up of 172 treated patients. Acta Derm. Venerol..

[25] Hassel J., Hassel A., Loser C. (2010). Phenol chemical matricectomy is less painful, with shorter recovery times but higher recurrence rates, than surgical matricectomy: A patient’s view. Dermatol. Surg..

[26] Shaikh F., Jafri M., Giri S., Keane R. (2008). Efficacy of wedge resection with phenolization in the treatment of ingrowing toenails. J. Am. Pod. Med. Assoc..

[27] Guerritsma-Bleeker C., Klaase J., Geelkerken R., Hermans J., van-Det R.J. (2002). Partial matrix excision or segmental phenolizationfor ingrowing toenails. Arch. Surg..

[28] Akkus A., Demirseren D.D., Demirseren M.E., Aktas A. (2018). The treatment of ingrown nail: Chemical matricectomy with NAOH versus wedge resection. Dermatol. Ther..

[29] Buckley D. (2000). Segmental phenolic ablation for ingrowing toenails in general practice. Ir. Med. J..

[30] Karaca N., Dereli T. (2012). Treatment of ingrown toenail with proximolateral matrix partial excision and matrix phenolization. Ann. Fam. Med..

[31] Morkane A., Robertson R., Inglis G. (1984). Segmental phenolization of ingrowing toenails: A randomized controlled study. Br. J. Surg..

[32] Pérez-Rey J., Mediavilla-Saldaña L., Martínez-Nova A. (2014). Exploring postoperative outcomes for ingrown toenails. NaOH vs Wedge resection techniques. Dermatol. Surg..

[33] Ozan F., Doğar F., Altay T., Uğur S.G., Koyuncu Ş. (2014). Partial matricectomy with curettage and electrocautery: A comparison of two surgical methods in the treatment of ingrown toenails. Dermatol. Surg..

